# CancerLocator: non-invasive cancer diagnosis and tissue-of-origin prediction using methylation profiles of cell-free DNA

**DOI:** 10.1186/s13059-017-1191-5

**Published:** 2017-03-24

**Authors:** Shuli Kang, Qingjiao Li, Quan Chen, Yonggang Zhou, Stacy Park, Gina Lee, Brandon Grimes, Kostyantyn Krysan, Min Yu, Wei Wang, Frank Alber, Fengzhu Sun, Steven M. Dubinett, Wenyuan Li, Xianghong Jasmine Zhou

**Affiliations:** 1 Molecular and Computational Biology, University of Southern California, Los Angeles, CA 90089 USA; 2 Department of Pathology and Laboratory Medicine, David Geffen School of Medicine, University of California at Los Angeles, Los Angeles, CA 90095 USA; 3 Institute for Quantitative and Computational Biosciences, University of California at Los Angeles, Los Angeles, CA 90095 USA; 4 Division of Pulmonary, Critical Care Medicine, Clinical Immunology and Allergy, David Geffen School of Medicine at UCLA, Los Angeles, CA 90095 USA; 5 VA Greater Los Angeles Healthcare System, Los Angeles, CA USA; 6 Department of Stem Cell Biology and Regenerative Medicine, and Norris Comprehensive Cancer Center, University of Southern California, Los Angeles, CA 90033 USA; 7 Clinical Laboratory, Zhejiang Province Tongde Hospital, Hangzhou, Zhejiang Province People’s Republic of China; 8 Department of Molecular and Medical Pharmacology, David Geffen School of Medicine, University of California, Los Angeles, CA 90095 USA; 9 Department of Medicine, David Geffen School of Medicine, University of California, Los Angeles, CA 90095 USA; 10 Jonsson Comprehensive Cancer Center, University of California, Los Angeles, CA 90095 USA

**Keywords:** Cell-free DNA, Liquid biopsy, DNA methylation, Next-generation sequencing, Cancer diagnosis

## Abstract

We propose a probabilistic method, CancerLocator, which exploits the diagnostic potential of cell-free DNA by determining not only the presence but also the location of tumors. CancerLocator simultaneously infers the proportions and the tissue-of-origin of tumor-derived cell-free DNA in a blood sample using genome-wide DNA methylation data. CancerLocator outperforms two established multi-class classification methods on simulations and real data, even with the low proportion of tumor-derived DNA in the cell-free DNA scenarios. CancerLocator also achieves promising results on patient plasma samples with low DNA methylation sequencing coverage.

**Electronic supplementary material:**

The online version of this article (doi:10.1186/s13059-017-1191-5) contains supplementary material, which is available to authorized users.

## Background

Cancer cells often display aberrant DNA methylation patterns, such as hypermethylation of the promoter regions of tumor suppressor genes and pervasive hypomethylation of intergenic regions [1–5]. Therefore, DNA methylation is an ideal target for cancer diagnosis in clinical practice [6, 7]. Hyper/hypomethylated tumor DNA fragments can be released into the bloodstream via cell apoptosis or necrosis, where they become part of the circulating cell-free DNA (cfDNA) in plasma [8]. The non-invasive nature of cfDNA methylation profiling makes it a promising strategy for general cancer screening. Current research on cfDNA-based, non-invasive cancer detection approaches falls into two classes: the development of biomarkers for a single specific cancer type; and the characterization of circulating tumor DNA (ctDNA) for general cancer detection, without trying to predict specific cancer types.

In recent years, several studies have reported plasma methylation biomarkers for different types of cancers [9–15]. Usually, the differentially methylated marker genes are identified by comparing methylation profile data from patients with a certain cancer type to healthy controls. However, these specific biomarkers are of limited use for general cancer screening. Ideally, as a non-invasive early screening tool, a liquid biopsy test should be able to detect many types of cancers and provide tumor location information for further specific clinical investigation.

Several approaches have recently been proposed for non-invasive universal cancer detection. These methods do not rely on detecting biomarkers specific to certain tumor types. Instead, they utilize properties of ctDNA that are common to various cancer types, such as copy number aberration (CNA) [16–19], pervasive hypomethylation [19], and DNA integrity [16, 20]. None of these methods can predict the tissue of origin after the detection of ctDNA. The nature of the liquid biopsy introduces a new challenge, in that the cancer type can remain unknown even when there is strong signal of tumor-derived DNA fragments in the blood. Hence, a positive result from a liquid biopsy would call for comprehensive follow-up investigations using clinical, analytical, and radiological tools to identify the tumor location. Considering that non-invasive screening is usually the first step of cancer diagnosis, and could be associated with a fair ratio of false positives, such follow-up would be likely to increase the burden on the medical care system. A few recent studies have proposed using cfDNA methylation [21, 22] or nucleosome footprinting [23] to partially alleviate this problem. For example, Sun et al. [21] estimated the proportions of cfDNAs contributed by different tissues and showed that an abnormally high proportion of cfDNA from a specific tissue can indicate the possibility of a tumor in that tissue. Their approach, though promising, has not been developed into a systematic method capable of supporting clinical diagnosis applications. Lehmann-Werman et al. [22] tested the same rationale to diagnose pancreatic cancer, but fewer than 50% of the pancreatic cancer patients demonstrated a substantial excess of pancreas-originated cfDNA fragments compared with healthy subjects. Snyder et al. [23] pioneered an approach of using nucleosome footprinting to predict the tissue of origin of the cfDNA, but its power in cancer diagnosis has not been demonstrated because only five plasma samples with high ctDNA burden were selected for testing from 44 late-stage cancer patients, and less than one half had their cancer types correctly predicted.

**Fig. 1:**
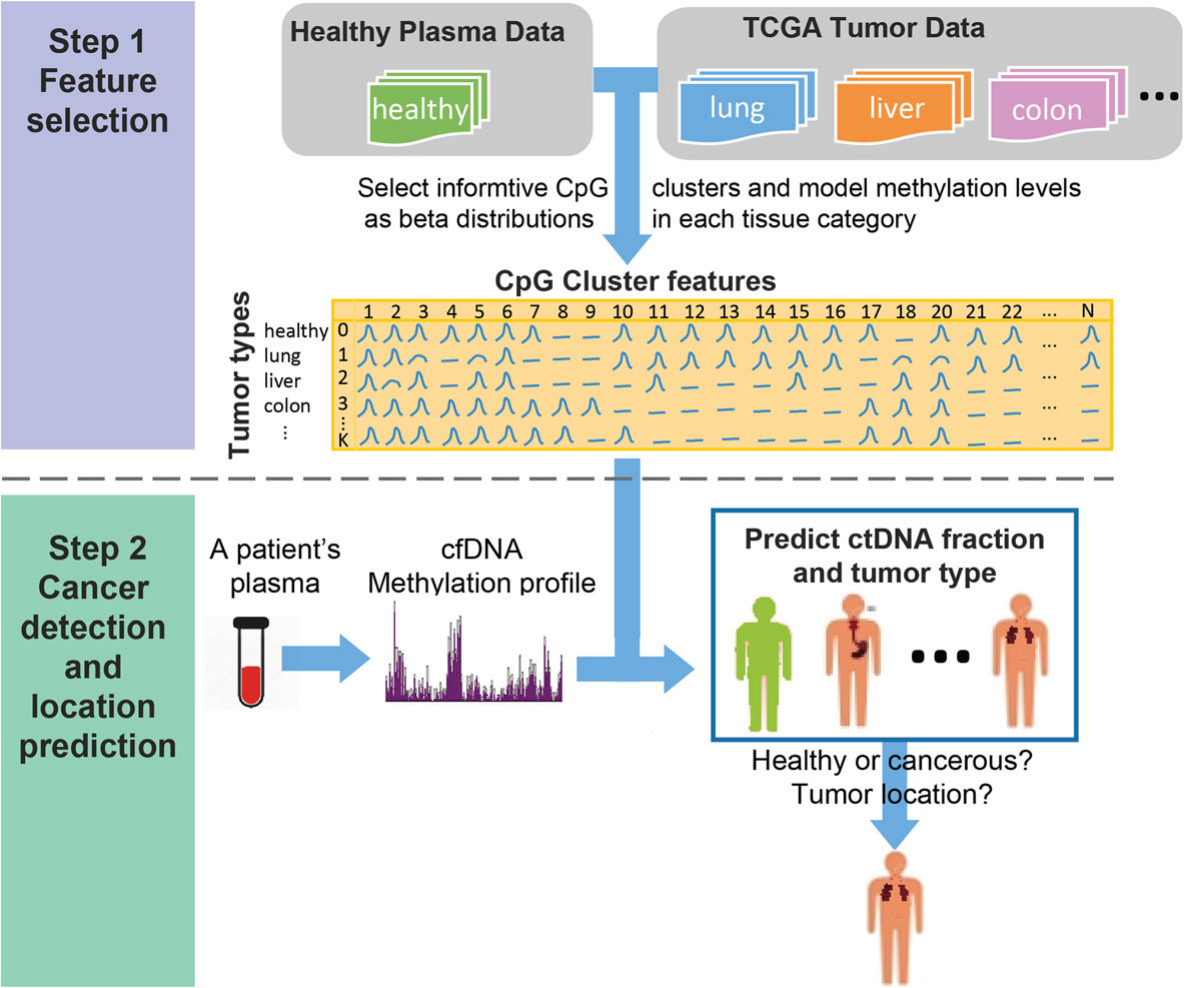
Flowchart of CancerLocator. Step 1: A set of solid tumor samples and healthy plasma samples collected from public databases and the literature are used to select the informative features (CpG clusters) that can differentiate tumor types or healthy plasma samples. Then the beta distributions of the methylation levels of these selected features for each tumor type or healthy plasma samples are learnt. Step 2: Given a plasma sample, the methylation profile of its cfDNAs is measured by whole-genome bisulfite sequencing, which is then used as input for cancer location prediction by CancerLocator

In summary, no existing cfDNA-based method can simultaneously detect cancer and predict its tissue of origin. We are therefore proposing a novel method, CancerLocator, that simultaneously infers the proportion and tissue of origin of ctDNA in a blood sample using genome-wide DNA methylation data. As shown in Fig. 1, from the vast amount of The Cancer Genome Atlas (TCGA) DNA methylation data, we first learn the informative features of different cancer types. We then model the plasma cfDNAs in cancer patients as a mixture of normal cfDNAs and ctDNAs. Finally, given the genome-wide methylation profile derived from the cfDNA sample of an unknown patient, CancerLocator uses the informative features to estimate the fraction of ctDNAs in the plasma and the likelihood that the detected ctDNAs come from each tumor type. Based on those likelihoods, CancerLocator makes the final decision on whether the patient has tumors and, if yes, the locations of the primary tumor.

We first evaluated our method on simulation data with known ctDNA fractions. The results show that CancerLocator can achieve a Pearson’s correlation coefficient (PCC) of 0.975 between the predicted and true proportions of ctDNA, and an error rate of 0.078 for the classification of non-cancer and tumor types. Moreover, our method far outperforms two well-established multi-class classification methods in both simulations and using real data, especially when the proportion of tumor-derived DNAs in the cfDNAs is lower than 50% (which is usually the case in reality). We note that CancerLocator achieved promising results on patient plasma samples, including around two-thirds of cancer samples collected from early-stage cancer patients.

## Results and discussion

### CancerLocator: a probabilistic method for predicting ctDNA burden and source tissue

A flowchart of CancerLocator is illustrated in Fig. 1. The first step is to identify the informative features of normal plasma and multiple tumor types from the massive TCGA database. We chose to focus on seven cancer types from the five organs (breast, colon, kidney, liver, and lung) that are generally regarded as having a high level of blood circulation. Given the plasma cfDNA methylation profile of a patient, the next step is to use those informative features to simultaneously detect cancer and locate its tissue of origin.

In the first step, we select CpG clusters (our procedure for grouping CpG sites into CpG clusters is described in the “Methods” section) as features if their methylation range (MR) is sufficiently large. MR is defined as the range of average methylation levels observed in healthy plasma and different solid tumor tissues. We selected *K* =14,429 CpG clusters (features), on average^1^, whose MRs are no less than the cutoff 0.25. For each CpG cluster, we take into account its variation across individuals by modeling the distribution of methylation levels for the same tumor type (or normal plasma) as a beta distribution, Beta(*α*_*t*_, *β*_*t*_). The index *t* = 0 represents normal plasma, while *t* = 1, …, *T* represents a tumor type.

In the second step, we use the selected features and their beta distributions to deconvolute a patient’s plasma cfDNA into the normal plasma cfDNA distribution and, possibly, a solid tumor DNA distribution. We have designed a probabilistic method that can simultaneously infer the burden and the tissue of origin of the ctDNA. Intuitively, if the likelihood of presence for any tumor type is not substantially higher than the likelihood that the observed distribution is the normal background, the patient is predicted to not have cancer. Otherwise, the patient is predicted to have the tumor type that is associated with the highest likelihood.

**Fig. 2:**
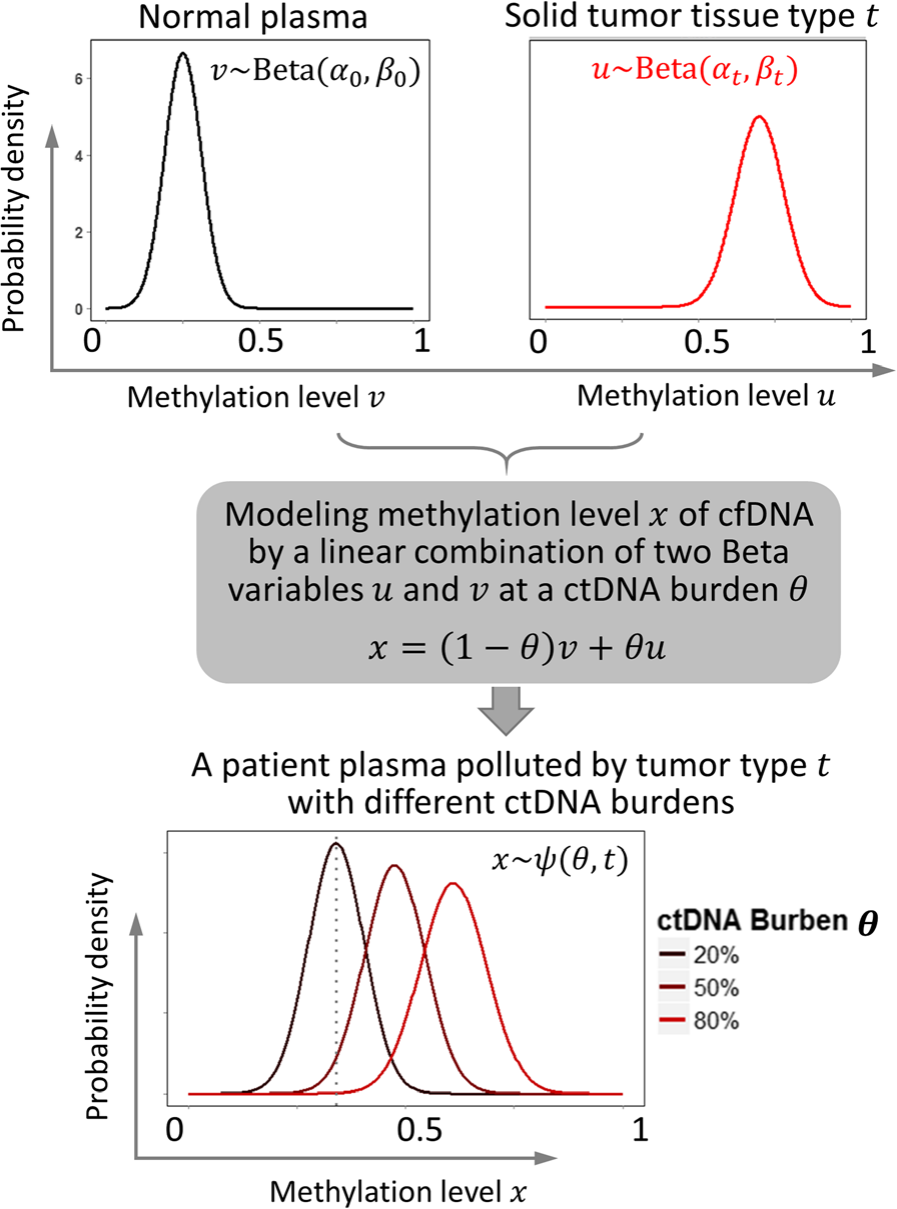
The mixture model of methylation level (*x*) in a patient’s plasma cfDNA for different burdens of ctDNAs from the tumor type *t*. Note that *x*, *u*, and *v* are the methylation levels of a single CpG cluster *k* in cfDNA, solid tumor, and normal plasma, respectively

Inferring the ctDNA burden *θ* and tumor type *t* can be formulated as a maximum-likelihood estimation (MLE) problem, where the likelihood function is expressed as the product of the likelihoods of each CpG cluster, assuming that all of the *K* selected CpG clusters are independent of each other. This is expressed as:

$$ L\left(\theta, t\Big| X\right)={\displaystyle \prod_{k=1}^K} L\left(\theta, t\Big|{x}_k\right) $$

where *x*_*k*_ denotes the methylation level of CpG site *k* in a cancer patient’s cfDNA. In principle, *x*_*k*_ is a linear combination of the DNA methylation levels in normal plasma and solid tumor type *t* with fraction *θ*. The normal and tumor components of the methylation are denoted by *v*_*k*_ and *u*_*k*_, respectively (Fig. 2). That is, *x* = (1 − *θ*)*v* + *θu* (for simplicity, we remove the subscript *k* from these notations). As mentioned earlier, since *v* and *u* follow the Beta distributions Beta(*α*_0_, *β*_0_) and Beta(*α*_*t*_, *β*_*t*_), respectively, *x* follows the distribution *ψ*(*θ*, *t*), which is calculated as the convolution of two Beta distributions Beta(*α*_0_, *β*_0_) and Beta(*α*_*t*_, *β*_*t*_).

Because cfDNA has low abundance in plasma, its methylation is usually measured by sequencing-based methods. Therefore, the methylation level *x*_*k*_ of CpG cluster *k* can be derived from two numbers, *n*_*k*_ and *m*_*k*_, denoting the total number of cytosines and the number of methylated cytosines mapped to CpG cluster *k*. We can model *m*_*k*_ and *n*_*k*_ together as a binomial distribution *m*_*k*_ ~ Binomial(*n*_*k*_, *x*_*k*_), and rewrite the likelihood function as:

$$ L\left(\theta, t\Big| M, N\right)={\displaystyle \prod_{k=1}^K} L\left(\theta, t\Big|{m}_k,{n}_k\right) $$

Detailed formulas and our optimization method are given in the “Methods” section.

For a comprehensive performance evaluation, we compare our method with two popular multi-class classification methods, i.e., random forest (RF) and support vector machine (SVM), on two types of data: simulation data with known ctDNA burden and real data with known clinical information but unknown ctDNA burden. The evaluations on simulation data and real data are complementary in assessing the predictive power of the methods.

### Prediction performance on the simulation data

The methylation data of a simulated plasma cfDNA sample is generated by computationally mixing the entire methylation profiles of a normal plasma cfDNA sample and a solid tumor sample (breast, colon, kidney, liver, or lung tumors), at a variety of ctDNA burdens (*θ* values). This strategy can make the simulated methylation data keep the potential correlations of methylation values between CpG clusters in real data. In addition, to make the simulated data more realistic, we add tumor CNA events at pre-defined probabilities (10, 30, and 50% across all CpG clusters). The procedure for these simulations is described in the “Methods” section. The results described below are on the simulation dataset with 30% CNA events—simulation data with other CNA event rates yield similar results (Additional file 1).

**Fig. 3:**
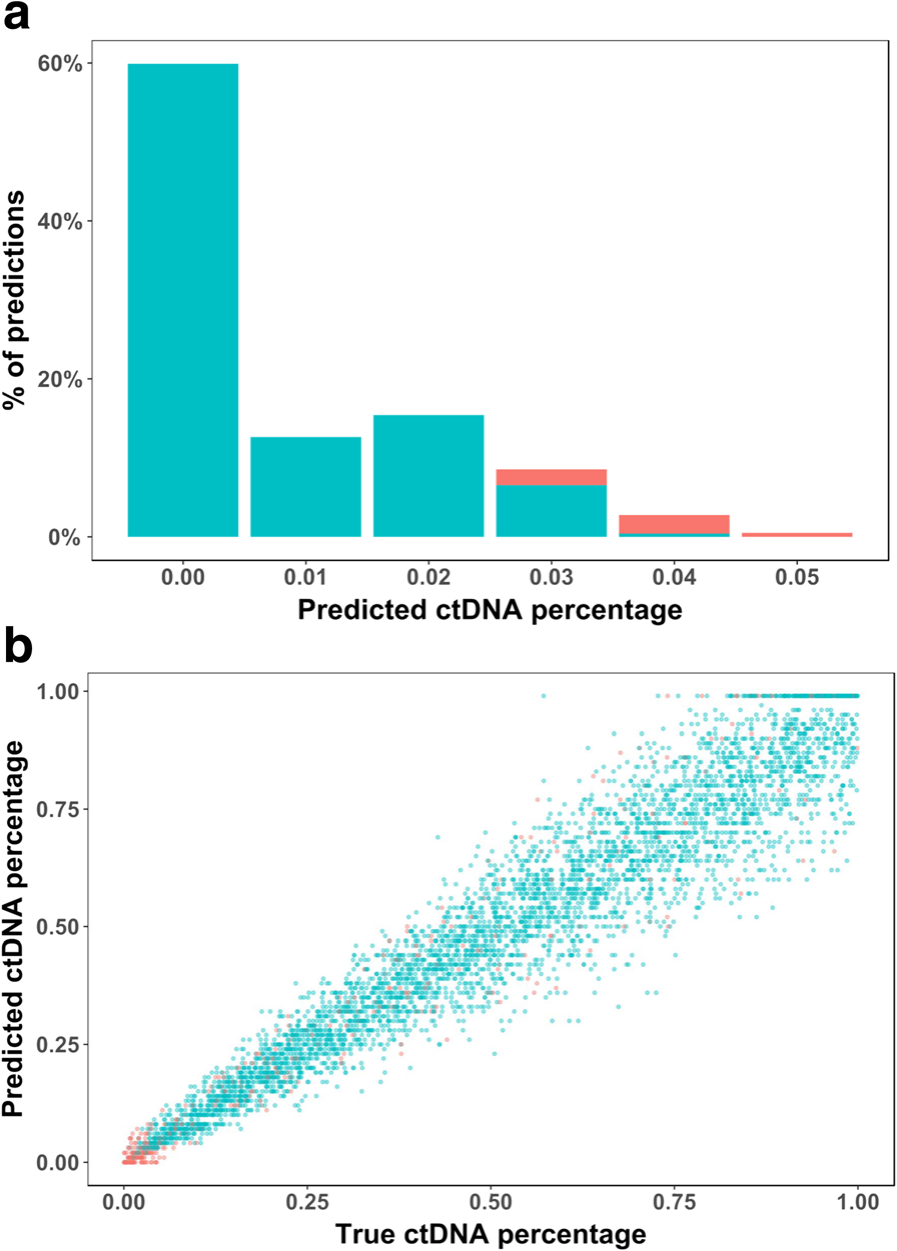
The predicted ctDNA burden for simulated normal and cancer plasma samples. **a** Predicted ctDNA burdens for normal samples whose true ctDNA burden should be zero. **b** Predicted and true ctDNA burdens for cancer samples. Each *dot* represents a prediction with the true (*x-axis*) and predicted (*y-axis*) ctDNA burdens. The correct and incorrect predictions are represented by *cyan* and *red*, respectively, in both **a** and **b**

We first assessed CancerLocator for ctDNA burden predictions. Overall, the predicted and true proportions of ctDNA are highly consistent, with a Pearson’s correlation coefficient of 0.975 and a root mean squared error of 0.074, respectively. As shown in Fig. 3a, the majority (87.9%) of the estimated ctDNA burdens for the normal samples are not more than 0.02, and none of them is greater than 0.05. Please note that whether a sample is from a cancer patient or not is determined by the optimal likelihood calculated in the prediction model, not the predicted ctDNA burden. The prediction results for the simulated cancer patient plasma samples are shown in Fig. 3b. We found that the variance of the predicted ctDNA burdens (*θ*) increases with the true *θ*, implying that the burden estimation becomes less precise when patients are in mid- or late cancer stages. This result could be partially explained by the fact that tumor heterogeneity may be higher in late stage tumor samples, which introduces the complexity of ctDNA burden prediction. However, this increased variance does not hurt the performance of the cancer detection because the predicted *θ* is still much higher than the normal background. Indeed, as demonstrated in Fig. 3b and below in the cancer type prediction results, the tissue origin of ctDNA becomes more distinguishable with high ctDNA burden, despite the increased variance in ctDNA prediction.

**Fig. 4:**
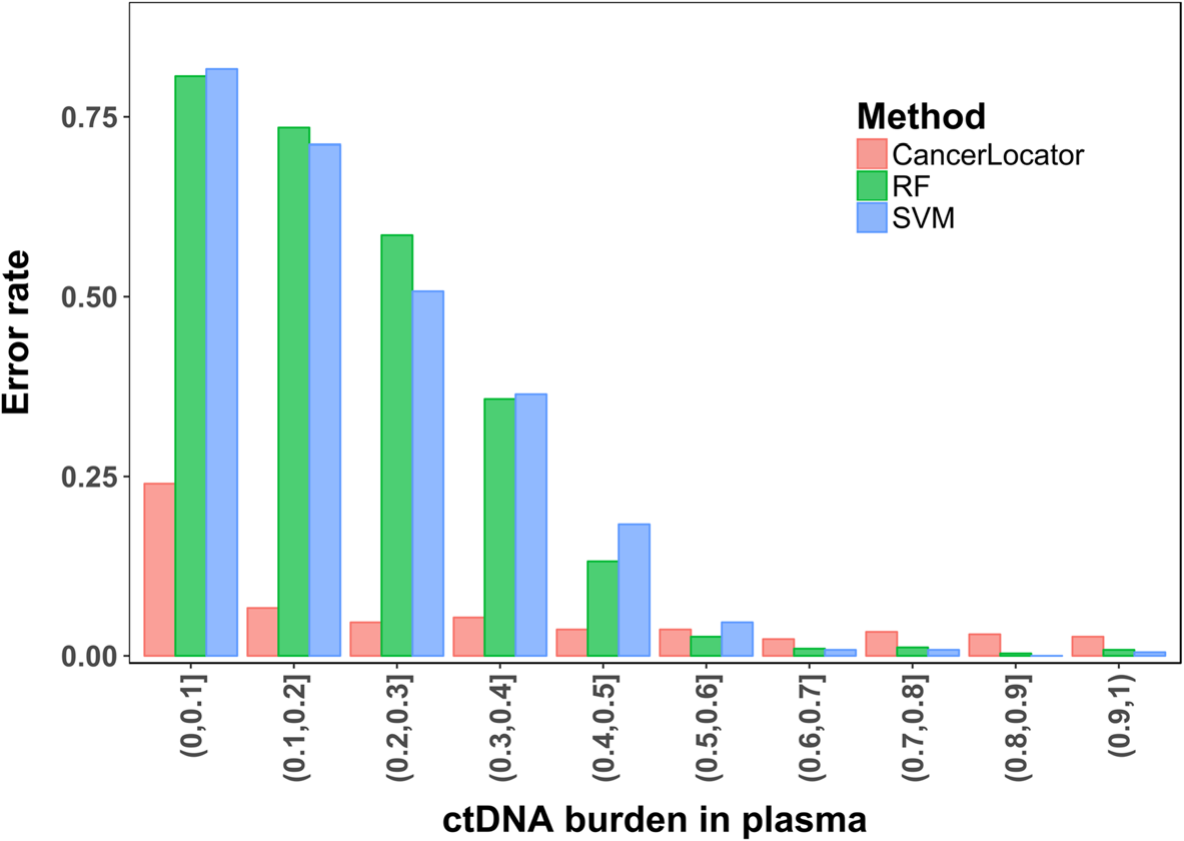
Classification performances of three methods (CancerLocator, RF and SVM) on the ten subsets of simulation data. Each subset includes plasma cfDNA samples at certain cancer stage (represented as a ctDNA burden range)

We then compared the performance of CancerLocator to that of two popular multi-class classification methods (RF and SVM; refer to Additional file 1 for details) using the same set of simulated samples. For a systematic comparison, we divided the simulation data into ten subsets for different cancer stages, each of which includes 200 normal plasma samples and 200 cancer plasma samples of each tumor type. The different cancer stages (from early, mid-, to late stages) are represented by a set of ctDNA burden ranges (*θ*, *θ* + 10%], where *θ* = 0, 10, 20, 30, 40, 50, 60, 70, 80, and 90%. For a six-class classification problem (normal, breast, colon, kidney, liver, and lung), we adopt the *error rate* measure for assessing the classification performance (see “Methods”). The results are shown in Fig. 4. For early-stage cancer patients with ctDNA burdens in the range *θ* ∈ (0, 10%], CancerLocator (error rate 0.240) largely outperforms RF and SVM (error rates 0.807 and 0.816, respectively), which are only slightly better than random guesses (0.833). For the second lowest ctDNA burdens *θ* ∈ (10%, 20%], CancerLocator reaches a very high prediction performance (error rate 0.067), while RF and SVM still have very poor performance (0.735 and 0.712, respectively). The two competing methods do not perform well until the ctDNA burdens are greater than 50%, which is mainly seen in plasma samples of late-stage cancer patients. The superior performance of CancerLocator on low to moderate ctDNA fractions indicates that without considering the mixture nature of cfDNAs in plasma, existing popular classification methods always fail to distinguish normal plasma samples and cancer patients’ plasma samples. This result highlights the advantage of our method for cancer diagnosis.

### Prediction performance on real plasma data

We randomly chose 75% of solid tumor samples and healthy plasma cfDNA samples as a training set to learn features. The remaining healthy plasma samples and all the cfDNA samples collected from cancer patients form the testing set, to which we applied CancerLocator, RF and SVM based on the selected features. After performing this procedure (including random data partition and predictions) ten times, the predictions of each of the three methods in ten runs were summarized into a confusion matrix, as shown in Table 1. Refer to the “Methods” section for detailed description of this procedure. For a new patient’s plasma sample, we assume that we have no prior information about the cancer type. Therefore, we also consider colon and kidney tumor as possible results, even though our real plasma data include no plasma samples from colon or kidney cancer patients.

**Table 1:** Confusion matrix of prediction results on the real plasma samples

Method	True class	Predicted class					
		Breast	Colon	Kidney	Liver	Lung	Non-cancer
CancerLocator	Breast	**20**	0	0	0	0	30
	Liver	0	0	20	**233**	33	4
	Lung	14	0	0	10	**68**	28
	Non-cancer	0	0	10	17	1	**142**
Random forest	Breast	0	0	1	0	1	48
	Liver	3	3	10	**53**	7	214
	Lung	4	0	1	0	**1**	114
	Non-cancer	0	0	0	1	0	**169**
SVM	Breast	0	0	0	0	15	35
	Liver	0	0	13	**66**	34	177
	Lung	0	0	1	0	**26**	93
	Non-cancer	0	0	1	0	12	**157**

Numbers in bold are correct predictions

The results in Table 1 show that our method vastly outperforms the two competing methods (RF and SVM). In fact, the competing methods cannot distinguish most cancer samples from non-cancer samples. Specifically, all the breast samples and the majority of liver and lung cancer samples are wrongly predicted as non-cancer by both RF and SVM. The overall error rates of RF and SVM are 0.646 and 0.604, respectively. In contrast, CancerLocator obtains a low error rate of 0.265 for the six-class prediction problem. These results are consistent with the simulation experiments for ctDNA burdens lower than 50%.

**Fig. 5:**
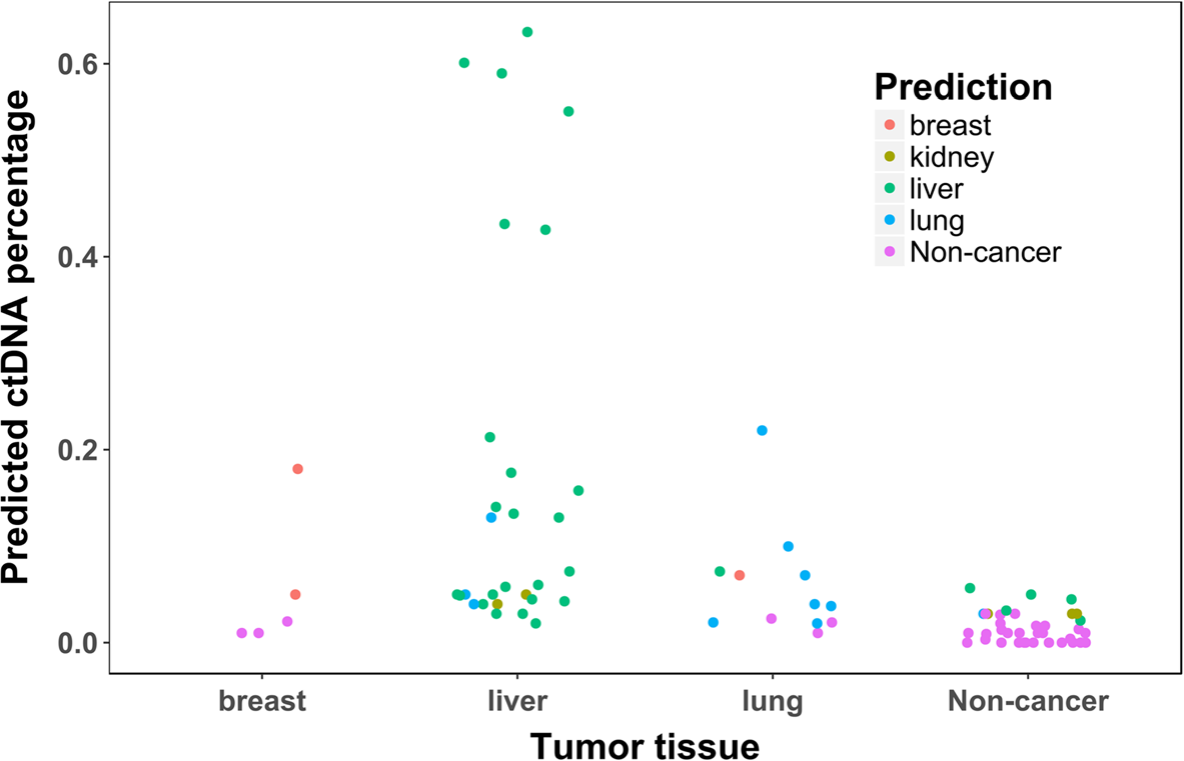
The relationship between ctDNA burden and tumor tissue prediction for each plasma sample of the real data. Each point represents a real plasma sample. This plot illustrates the average estimated tumor burden (*y-axis*) and the most frequently predicted tumor type (*dot color*) among ten runs for each plasma sample

To understand the relationship between estimated ctDNA burdens and tumor types in real data, we plotted their relationships in Fig. 5 by summarizing predictions for each plasma sample in all ten runs: the average estimated ctDNA burden (y-axis value) and the most frequently predicted tumor type (dot color) among ten runs for each sample. It can be observed that the higher the estimated ctDNA burden, the more accurate the prediction of tumor type. This is highly consistent with the results from the simulation data. For the breast cancer samples, three out of five samples have ctDNA burdens ≤2.2%, and they are all predicted as non-cancer. The inferred tumor burden of the two correctly predicted samples are 5.0 and 18.0%, respectively, and the latter is a metastatic sample. For the 29 liver cancer samples, at least 25 of them are from early-stage (Barcelona Clinic Liver Cancer stage A) patients. Most of them (80%) were classified as liver cancer and all of them were detected as cancer samples. Compared to the breast cancer samples, most of the liver samples, even at an early stage, can have moderate to high tumor burden (average predicted tumor burden of 14.9% and the highest reaching 59.0%), given that liver has generally excellent blood circulation, but we also correctly classified the one with only 2.0% predicted tumor burden as liver cancer. Among the 12 lung cancer samples (two samples did not have cancer stage information), at least five were collected from early-stage patients. These early-stage samples have predicted tumor burdens ranging from 2.0 to 4.0%. Among these five early-stage lung cancer samples, four were correctly predicted as lung cancer, whereas the remaining one was predicted as non-cancer.

We also note that CancerLocator correctly predicted seven out of eight chronic hepatitis B virus (HBV) samples to be non-cancer samples. In addition, our method successfully predicted the only one sample with benign lung tumor as non-cancer in all ten runs, with the predicted ctDNA burden always being 0.0%. These results demonstrate that CancerLocator can go beyond distinguishing healthy samples from cancer samples and handle more sophisticated scenarios, such as differentiating HBV carriers or benign tumor patients from cancer patients.

## Conclusions

Blood-based cancer diagnosis, unlike traditional diagnosis based on tissue biopsy, has the potential to diagnose tumors from many organs. The proposed CancerLocator aims to exploit this potential of cfDNA by not only diagnosing the presence of tumors, but also predicting the tissue of origin. Although three very recent studies have investigated the inference of tissue of origin [21–23], these works lack either a well-developed prediction method [21] or systematic performance evaluations [22, 23]. Unlike these previous studies, we lay out a systematic prediction method for cfDNA-based cancer type inference, comprehensively evaluate its performance on both simulated data and real data, and compare its performance to that of two established multi-class classification methods. We show that having a mixture of plasma cfDNAs can completely defeat standard machine learning methods for cancer type predictions when the proportion of tumor-derived DNA is lower than 50%. In contrast, CancerLocator successfully overcomes this obstacle. The poor performance of the standard methods is largely caused by their treatment of the samples in each tumor class as independent and identically distributed, following some class-specific distribution, while in our model the samples from the same class can still be very different due to different ctDNA percentages in the blood. In addition, our results show that our method is robust to CNA events, possibly because the genome-wide features outweigh the local aberrations.

In this work, we used DNA methylation microarrays of solid tumor tissues to train the model due to the scarcity of whole-genome bisulfite sequencing data (WGBS) in the public domain. Since DNA methylation arrays focus only on promoter regions, they may miss important signature regions of cancer. Therefore, we expect that the growing amount of WGBS data will significantly empower the proposed approach by revealing better and higher resolution signatures. Owing to the limited number of plasma samples, the results of this study are evaluated only on three cancer types (breast, liver and lung). However, our new approach has the potential to perform well on all cancer types with well-circulated originating organs. Also, due to the limited plasma samples, the cutoff of the prediction score *λ* (defined in the “Methods” section and computed based on the likelihood) used to differentiate cancer or non-cancer samples is specifically determined for this set of plasma samples for the best performance. When data on more plasma samples become available, this cutoff could be determined by the training data to be robust to most testing scenarios. Finally, we note that we identified markers by comparing methylation profiles of normal plasma cfDNAs and tumor DNAs. This procedure may introduce markers that are tissue-specific but not tumor-specific. This effect can be largely reduced by first using paired samples (tumor sample and the matched adjacent non-tumor sample) to identify tumor-specific markers, then further narrowing down to those markers that show differentiating signals from normal plasma cfDNAs. We foresee the increased power by such identified biomarkers when sufficient paired samples become available.

## Methods

In this section, we describe: 1) how the data are processed (including methylation microarray and sequencing data); 2) the implementation of CancerLocator; 3) how the simulation data are generated while taking into account copy number aberrations; 4) how the training and testing data are split; and 5) what measures we use to evaluate performance.

### Methylation data collection and processing

#### Data collection

We collect a large set of public methylation data of solid tumors and plasma cfDNA samples taken from both healthy people and cancer patients. The majority of tumor methylation profiles in TCGA were assayed using the Infinium HumanMethylation450 microarray. We collect those data for solid tumors with >100 samples from five different organs: 681 samples of breast (BRCA), 290 samples of colon (COAD), 522 samples of kidney (including 300 KIRC and 156 KIRP samples), 169 samples of liver (LIHC), and 809 samples of lung (including 450 LUAD and 359 LUSC samples) cancer.^2^

The public methylation data of plasma cfDNA samples are from Chan et al. [19] and Sun et al. [21]. The two datasets include the WGBS data of plasma samples taken from 32 normal people, eight patients infected with HBV, 29 liver cancer patients, four lung cancer patients, five breast cancer patients, and a number of patients with tumors in organs without a large blood flow. We also generated WGBS data from plasma samples collected from eight cancer patients (five early-stage lung cancer patients, one late-stage lung cancer patient, two lung cancer patients with unknown stage information) and one patient with a benign lung tumor. We used only the normal, HBV, and breast/liver/lung cancer patients in our study, for a total of 87 plasma samples. Note that these public WGBS data have very low sequencing coverage (~4× on average), while the coverage of our newly generated data for all nine samples is around 10×.

#### Human subjects

The blood samples of eight lung cancer patients and one benign lung tumor patient were collected. The demographic and clinical features of the patients profiled are presented in Additional file 1: Table S2.

#### Cell-free DNA isolation and whole-genome bisulfite sequencing

Blood samples were centrifuged at 1600 × g for 10 minutes and then the plasma was transferred into new microtubes and centrifuged at 16,000 × g for another 10 minutes. The plasma was collected and stored at −80 °C. cfDNA was extracted from 5 ml plasma using the Qiagen QIAamp Circulating Nucleic Acids Kit and quantified using a Qubit 3.0 Fluoromter (Thermo Fisher Scientific). Bisulfite conversion of cfDNA was performed using a EZ-DNA-Methylation-GOLD kit (Zymo Research). After that, an Accel-NGS Methy-Seq DNA library kit (Swift Bioscience) was used to prepare the sequencing libraries. The DNA libraries were then sequenced with 150-bp paired-end reads.

#### Building features (CpG clusters)

The Infinium HumanMethylation450 microarray data from TCGA measure all solid tumor samples at ~450,000 CpGs. Since our testing sample [19] comprises WGBS data with very low sequencing coverage, we grouped the CpG sites into CpG clusters in order to use more mappable reads. For a CpG site covered by a probe on the microarray, we define the region 100 bp up- and downstream as its flanking region and assume that all CpG sites located within this region have the same average methylation level as the CpG sites covered by probes. Two adjacent CpG sites are grouped into a CpG cluster if their flanking regions overlap. Finally, only those CpG clusters containing at least three CpGs covered by microarray probes are used in this study. We choose the size of the flanking region and the number of CpGs in a cluster according to three criteria: (i) at least three CpG sites (in the microarray data) are included to obtain a robust measurement of methylation values in the solid tumor samples; (ii) the cluster is reasonably sized, so that there are sufficient CpG sites to calculate the methylation values, even when low coverage sequencing data are used; (iii) keep as many clusters that span within a type of genomic region (either CpG islands or shores) as possible. This procedure yielded 42,374 CpG clusters, which together include about one-half of all the CpG sites on the Infinium HumanMethylation450 microarray. Most of these clusters are each associated with only one gene. These CpG clusters are used for subsequent feature selection.

#### Methylation microarray data processing

The microarray data (level 3 in TCGA database) provide the methylation levels of individual CpG sites. We define the methylation level of a CpG cluster as the average methylation level of all CpG sites in the cluster. A cluster’s methylation level is marked as “not available” (NA) if more than half of its CpG sites do not have methylation measurements.

#### WGBS data processing

Bismark [24] is employed to align the reads to the reference genome HG19 and call the methylated cytosines. After the removal of PCR duplications, the numbers of methylated and unmethylated cytosines are counted for each CpG site. The methylation level of a CpG cluster is calculated as the ratio between the number of methylated cytosines and the total number of cytosines within the cluster. However, if the total number of cytosines in the reads aligned to the CpG cluster is less than 30, the methylation level of this cluster is treated as NA.

#### Feature filtering

For each CpG cluster, we used the methylation range (MR) to indicate a feature’s differential power between classes. We first obtained the average methylation level of all samples from each class (i.e., healthy plasma or each tumor type), then defined MR as the range of this set of mean values (i.e., the difference between the largest and smallest mean values). The higher the MR of a cluster is, the more differential power it has. Finally, we selected those CpG clusters whose MRs were no lower than a threshold.

### Statistical inference of the ctDNA burden and tissue of origin

#### A mixture model of methylation levels of plasma cfDNAs

The cfDNA in the plasma of cancer patients can be regarded as a mixture of normal background DNA and tumor-released DNA. Formally, for each CpG cluster *k* ∈ {1, 2, ⋯, *K*}, the methylation level *x*_*k*_ of the plasma cfDNA from a given patient can be approximated as a mixture of *v*_*k*_ and *u*_*k*_, which are the methylation levels of the normal plasma sample and the solid tumor tissue, respectively. Let *θ* ∈ (0, 1) denote the proportion of tumor-derived DNAs in plasma cfDNA. Then *x*_*k*_ can be expressed as the weighted sum of *v*_*k*_ and *u*_*k*_, i.e., *x*_*k*_ = (1 − *θ*)*v*_*k*_ + *θu*_*k*_.

We assume that an individual carries at most one type of tumor among the *T* possible tumor types. Let *t* ∈ {0, 1, 2, ⋯, *T*} be the variable representing either normal plasma (*t* = 0) or a tumor type (1 ≤ *t* ≤ *T*). For each CpG cluster *k*, we model its methylation level in a sample of type *t* as a Beta distribution: *v*_*k*_ ~ Beta(*α*_*k*0_, *β*_*k*0_) for normal plasma samples (*t* =0) and *u*_*k*_ ~ Beta(*α*_*kt*_, *β*_*kt*_) for solid tumor samples of type *t* ∈ {1, ⋯, *T*}, where *α*_*k*0_ and *β*_*k*0_ (*α*_*kt*_ and *β*_*kt*_) are the parameters of the beta model of methylation levels of CpG cluster *k* in normal plasma (solid tumor) samples. As illustrated in step 1 of Fig. 1, the parameters of these Beta distributions are estimated by the method of moments, using the large amount of public tumor data and normal plasma data.

By integrating the two Beta distributions (*v*_*k*_ and *u*_*k*_), as shown in Fig. 2, *x*_*k*_ can be modeled by a derived distribution with the given ctDNA burden *θ* and source tumor type *t*. This model is denoted as the probability density function *ψ*(*x*_*k*_|*θ*, *t*), which is calculated by the convolution of Beta(*α*_*k*0_, *β*_*k*0_) and Beta(*α*_*kt*_, *β*_*kt*_). It is formally expressed as:

1 $$ \psi \left({x}_k\Big|\theta, t\right)={\displaystyle \underset{0}{\overset{1}{\int }}}{f}_{\mathrm{Beta}}\left(\frac{x_k-\theta {u}_k}{1-\theta}\Big|{\alpha}_{k0},{\beta}_{k0}\right){f}_{\mathrm{Beta}}\left({u}_k\Big|{\alpha}_{k t},{\beta}_{k t}\right)\; d{u}_k $$

where *f*_Beta_ is the probability mass function of the Beta distribution.

#### Modeling the methylated cytosine count of plasma cfDNA sequencing data

Due to its low abundance in plasma, the methylation profile of cfDNA is usually measured by sequencing-based methods, and the methylation levels (*x*_*k*_) of a CpG cluster *k* can be characterized by the numbers of methylated and unmethylated cytosines in the reads. Let *M* = (*m*_1_, *m*_2_, ⋯, *m*_*K*_) and *N* = (*n*_1_, *n*_2_, ⋯, *n*_*K*_) be the number of methylated cytosines and the total number of cytosines mapped to all CpG sites, respectively, where the index runs over all *K* CpG clusters. For each CpG cluster *k*, *m*_*k*_ can be modeled by a binomial distribution: *m*_*k*_ ~ Binomial(*n*_*k*_, *x*_*k*_). By integrating the mixture model of *x*_*k*_ in Eq. 1, we have the likelihood function for each CpG cluster *k* which has the inputs from the model parameters (*θ*, *t*, *α*_*k*0_ and *β*_*k*0_, *α*_*kt*_, and *β*_*kt*_) and the sequence measurements of plasma samples (*m*_*k*_, *n*_*k*_):

2 $$ f\left({m}_k\Big|\theta, t,{n}_k\right)={\displaystyle \underset{0}{\overset{1}{\int }}}{f}_{\mathrm{Binomial}}\left({m}_k\Big|{n}_k,{x}_k\right)\psi \left({x}_k\Big|\theta, t\right)\; d{x}_k $$

where *f*_Binomial_ is the probability density function of the binomial distribution.

#### Maximum-likelihood estimation of blood tumor burden and type

Given the methylation sequencing profile of a patient’s plasma cfDNA sample, the vectors *M* and *N*, we aim to find the maximum-likelihood estimate of two model parameters: a sample’s cfDNA tumor burden *θ* and its source tumor type *t*. For integrating the mixture models of multiple markers into the formulation, we adopted a commonly used assumption: all features or markers are independent of each other. This assumption has been widely used in a number of cell-type deconvolution studies [25, 26]. Under this assumption, the log-likelihood can be written as:

3 $$ \log \kern0.5em  L\left(\theta, t\Big| M, N\right)={\displaystyle \sum_{k=1}^K}\kern0.5em  \log \kern0.5em  f\left({m}_k\Big|\theta, t,{n}_k\right) $$

Since the integrals in Eqs. 1 and 2 cannot be easily solved analytically, we use Simpson's rule to calculate the log-likelihood. That is, a set of *J* predefined *θ* values, $\Theta =\left\{0,\frac{1}{J},\frac{2}{J},\dots, \frac{J-1}{J}\right\}$, is used to conduct a grid search for the best estimation (i.e., a global optimization solution). The higher the resolution (*J*), the more precise the estimation. After obtaining the solution (i.e., $\hat{\theta}$ and $\hat{t}$) that maximizes Eq. 3, we use the estimated parameters to calculate a simple yet effective prediction score that answers two questions: “Does the patient have cancer?”; and “If the patient has cancer, which tumor type is it?” This prediction score is defined below:

4 $$ \lambda =\frac{1}{K}\left[ \log \kern0.5em  L\left(\hat{\theta},\hat{t}\Big| M, N\right)- \log \kern0.5em  L\left(\theta =0\Big| M, N\right)\right] $$

where the denominator *K* is used to normalize the log-likelihood, so that *λ* is comparable when using a different number of features. The variable *t* is not included in *L*(*θ* = 0|*M*, *N*) because *θ* = 0 indicates a normal plasma sample. The larger the prediction score *λ*, the higher the chance that the patient has a cancer tumor of type $\hat{t}$. Specifically, if *λ* is greater than a threshold, the patient is predicted as having cancer with the ctDNA burden $\hat{\theta}$ and the tumor type $\hat{t}$; otherwise, he/she is classified as not having cancer.

### Simulation data generation

We simulate the methylation sequencing data of a patient’s plasma cfDNAs using the previously described probabilistic models: (i) a mixture model that treats the cfDNA as a mixture of normal plasma cfDNA and DNAs released from primary tumor sites; and (ii) a binomial model for the methylated cytosine count of plasma cfDNA sequencing data. In addition, to make the simulation data more realistic, we incorporate CNAs and read depth bias. The procedure for simulating plasma cfDNA methylation sequencing data is detailed in the following sections.

#### Inputs

Inputs include: (i) the genomic regions of all *K* CpG clusters; (ii) the total number of cytosines (Z) on the sequencing reads that are aligned to any CpG cluster; (iii) the range of *θ* : (*θ*_*L*_, *θ*_*U*_); (iv) the collections of normal plasma samples (denoted as POOL_normal_) and solid tumor samples (denoted as POOL_tumor_); and (v) *b*_*k*_, the background probability for a CpG dinucleotide to be aligned to CpG cluster *k*, satisfying ∑_*k* = 1_^*K*^ *b*_*k*_ = 1. The last input reflects the read-depth bias introduced during the sequencing process and read alignment and the density of CpG sites in the clusters. Refer to Additional file 1 for details of how to obtain *b*_*k*_.

#### Output

Output comprises a simulated methylation sequencing profile of a plasma sample, represented by the integer vectors *M* = (*m*_1_, *m*_2_, ⋯, *m*_*K*_) and *N* = (*n*_1_, *n*_2_, ⋯, *n*_*K*_). The elements *m*_*k*_ and *n*_*k*_ are the number of methylated cytosines and the total number of cytosines in the reads mapped to CpG cluster *k*, respectively.

### Procedure

1. Generate a random ctDNA fraction *θ* from the distribution *θ* ~ Uniform(*θ*_*L*_, *θ*_*U*_).
2. Generate a random integer copy number *c*_*k*_ for each CpG cluster *k*, from the categorical distribution *c*_*k*_ ~ Cat(6, *p*_0_, *p*_1_, *p*_2_, *p*_3_, *p*_4_, *p*_5_). Here, *p*_*c*_ denotes the probability of observing copy number *c* ∈ {0, 1, 2, 3, 4, 5} in the sequencing data. The probabilities *p*_*c*_ satisfy three criteria: (i) their sum is equal to one, $$ \begin{array}{l}{\displaystyle {\sum}_{\mathrm{c}=0}^5{\mathrm{p}}_{\mathrm{c}}=1}\\ {}\end{array} $$; (ii) the average copy number is equal to two, ∑_c = 0_^5^c ∗ p_c_ = 2; and (iii) extreme CNAs are less likely to occur. In this work, we predefine *p*_0_ = 0.005, *p*_1_ = 0.16, *p*_2_ = 0.7, *p*_3_ = 0.105, *p*_4_ = 0.025, *p*_5_ = 0.005. Note that the sum of all these probabilities except *p*_2_ (30% in this case) is the probability of any given CpG cluster having a CNA event. We have tried other probability configurations for the simulation with more (50%) or fewer (10%) CNA events and obtained similar results (Additional file 1). No CNA event is considered (i.e., *c*_*k*_ is fixed to two) when simulating a normal plasma sample.
3. Randomly select a normal plasma sample from POOL_normal_ whose methylation profile is denoted by (*v*_1_, *v*_2_, ⋯, *v*_*K*_), and randomly select a solid tumor from POOL_tumor_ whose methylation level profile is denoted by (*u*_1_, *u*_2_, ⋯, *u*_*K*_). Note that we also randomly select two normal plasma samples from POOL_normal_ in order to simulate a new normal plasma sample.
4. Calculate the methylation level *x*_*k*_ of plasma cfDNA at CpG cluster *k*. This is the adjusted linear combination of *v*_*k*_ and *u*_*k*_ after incorporating the copy number *c*_*k*_ generated in step 2. That is, *x*_*k*_ = (1 − *θ*_*k*_^'^)*v*_*k*_ + *θ*_*k*_^'^ *u*_*k*_, where *θ*_*k*_^'^ is the adjusted value of *θ* given by ${\uptheta}_{\mathrm{k}}^{\hbox{'}}=\frac{{\uptheta \mathrm{c}}_{\mathrm{k}}}{{\uptheta \mathrm{c}}_{\mathrm{k}}+2\left(1-\uptheta \right)}$. *θ*_*k*_^'^ describes the actual ctDNA fraction after considering the copy number *c*_*k*_ of the ctDNA.
5. Generate a random number *n*_*k*_, representing the total number of cytosines in CpG cluster *k*, from the Poisson distribution *n*_*k*_ ~ Poisson(*ZB*_*k*_). *B*_*k*_ is the adjusted CpG dinucleotide bias *b*_*k*_, given by ${\mathrm{B}}_{\mathrm{k}}=\frac{{\mathrm{b}}_{\mathrm{k}}\left(1-\uptheta +{\uptheta \mathrm{c}}_{\mathrm{k}}/2\right)}{{\displaystyle {\sum}_{\mathrm{k}=1}^{\mathrm{K}}}{\mathrm{b}}_{\mathrm{k}}\left(1-\uptheta +{\uptheta \mathrm{c}}_{\mathrm{k}}/2\right)}$, after scaling with the copy number *c*_*k*_ generated in step 2.
6. Generate a random number *m*_*k*_ from the binomial distribution *m*_*k*_ ~ Binomial(*n*_*k*_, *x*_*k*_).

Due to the limited number of normal plasma samples, we also simulated new normal plasma samples by mixing two normal plasma samples at different mixture ratios. The procedure is the same as above except that step 2 is ignored by fixing all copy numbers as two because there are no CNA events in the normal plasma samples.

### Performance evaluation

#### Data partitions for learning signature features, simulation, and real data experiments

**Fig. 6:**
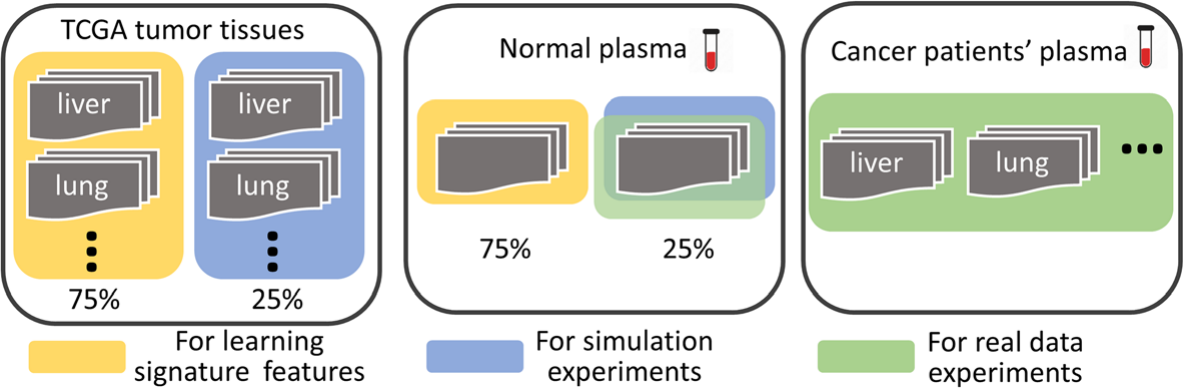
Illustration of the data partition for learning discriminating features, in both simulation and real data experiments. Note that simulation and real data experiments share the same subset (25%) of normal plasma samples

All TCGA solid tumor tissues and plasma samples are divided into non-overlapping sets for three tasks: (i) learning discriminating features; (ii) simulation experiments; and (iii) testing on the real data. Specifically, as shown in Fig. 6, we split TCGA solid tumors of each tissue type into two partitions: 75% for learning signature features and 25% for generating simulation data. We also split all normal plasma samples into two partitions: 75% for learning signature features and 25% for generating simulation data or for real data experiments. All the plasma samples of the cancer patients are used to form the testing set in the real data experiments. Note that not these plasma samples, but only solid tumor samples collected from public methylation databases, and a subset of normal plasma samples that were not used for testing, were used for learning features. All data are randomly partitioned following the above proportions, and applying a method on one such partition is regarded as “one run”. For making the robust results, we repeat the experiments for ten runs and aggregate all predictions obtained in the ten runs into a single confusion matrix as the final result. Because we had a limited number of real cancer plasma samples (only 5, 12, and 29 cfDNA samples from breast, lung, and liver cancer patients, respectively) for testing, it would not allow the typical cross-validation for the method’s hyperparameter estimation. For fully utilizing the test samples for effective performance evaluation, we report only the best prediction results for each of three methods (CancerLocator, RF and SVM) after examining all possible values of each method’s hyperparameters. The only hyperparameter of CancerLocator is the threshold of the prediction score *λ*, which is set as 0.023 to generate the predictions on the real plasma samples. For consistency with the real data experiments, we apply the same strategies to simulation data experiments and calculate the error rate averaged over ten runs.

#### Prediction performance measures

The error rate and accuracy are the most popular and established multi-class classification performance measures [27–29]. They are equivalent to each other. This study uses the error rate, which is defined as the percentage of incorrect predictions out of all predictions.

## Additional file

Additional file 1: Supplementary information. A PDF file including Figures S1 and S2, Tables S1 and S2, as well as the details of background bias estimation of CpG read counts, the RF and SVM methods, and CancerLocator’s prediction results on simulation data with different levels of CNA events. (PDF 558 kb)

## Footnotes

1 We randomly select a subset of the normal plasma and TCGA tumor samples for training and use the rest to simulate samples for testing. This procedure is repeated ten times. Different training sets may lead to different numbers of selected CpG clusters. We therefore report the average number of features here. Our data partition strategy is illustrated in Fig. 6 and described in the “Methods” section.

2 BRCA, Breast invasive carcinoma; COAD, Colon adenocarcinoma; KIRC, Kidney renal clear cell carcinoma; KIRP, Kidney renal papillary cell carcinoma; LIHC, Liver hepatocellular carcinoma; LUAD, Lung adenocarcinoma; LUSC, Lung squamous cell carcinoma.
