# Supplementary material for: CancerLocator: non-invasive cancer diagnosis and tissue-of-origin prediction using methylation profiles of cell-free DNA
Source: Genome Biol. 2017 Mar 24;18:53. doi: 10.1186/s13059-017-1191-5 (PMC5364586; doi:10.1186/s13059-017-1191-5)
Supplement: Additional file 1: — Supplementary information. A PDF file including Figures S1 and S2, Tables S1 and S2, as well as the details of background bias estimation of CpG read counts, the RF and SVM methods, and CancerLocator’s prediction results on simulation data with different levels of CNA events. (PDF 558 kb) [file 13059_2017_1191_MOESM1_ESM.pdf]

Supplemental Information for CancerLocator: Non-invasive cancer diagnosis and tissue-of-origin prediction using methylation profiles of cell-free

Shuli Kang[1], Qingjiao Li[1], Quan Chen[1], Yonggang Zhou[2,3], Stacy Park[4], Gina Lee[5], Brandon Grimes[4], Kostyantyn Krysan[4], Min Yu[6], Wei Wang[7], Frank Alber[1], Fengzhu Sun[1], Steven M. Dubinett[2,8,9,10] *, Wenyuan Li[2] *, Xianghong Jasmine Zhou[2,3] *

1. Molecular and Computational Biology, University of Southern California, Los Angeles, CA 90089, USA

2. Department of Pathology and Laboratory Medicine, David Geffen School of Medicine, University of California at Los Angeles, CA 90095, USA

3. Institute for Quantitative and Computational Biosciences, University of California at Los Angeles, CA 90095, USA

4. Division of Pulmonary, Critical Care Medicine, Clinical Immunology and Allergy, David Geffen School of Medicine at UCLA, Los Angeles, CA 90095, USA

5. VA Greater Los Angeles Healthcare System, Los Angeles, CA, USA

6. Department of Stem Cell Biology and Regenerative Medicine, and Norris Comprehensive Cancer Center, University of Southern California, Los Angeles, CA 90033, USA

7. Clinical Laboratory, Zhejiang Province Tongde Hospital, Hangzhou, Zhejiang Province, People's Republic of China

8. Department of Molecular and Medical Pharmacology, David Geffen School of Medicine, University of California, Los Angeles, Los Angeles, CA 90095, USA

9. Department of Medicine, David Geffen School of Medicine, University of California, Los Angeles, Los Angeles, CA 90095, USA

10. Jonsson Comprehensive Cancer Center, University of California, Los Angeles, Los Angeles, CA 90095, USA

*Co-corresponding authors

A. Background bias estimation of CpG read counts

The bias of CpG read counts in the WGBS data is presented as a vector $B = \{b_1, b_2, ..., b_K\}$, where each element in the vector is the probability that the CpG dinucleotides on the sequencing reads coming from corresponding CpG cluster. To estimate B, we merge the WGBS data of normal buffy coat samples reported by Chan *et al*. [1], and calculate b_i as the percentage of the CpG dinucleotides on the reads mapped to cluster i, among those mapped to any of the clusters.

B. Details of random forest and support vector machine

The two competing methods, random forest (RF) and support vector machine (SVM), are implemented using the popular Python package "scikit-learn" [2]. To explore the potential of the two methods, features (CpG clusters) used in the two methods are selected according to their "importance", which can be measured by the output or by-product of a multiclass prediction model (either random forest or support vector machine). Specifically, we adopt both of the following popular feature importance measure:

1. The "***gini feature importance***", which is output of **random forest (RF)** [3,4]. It is defined as the total decrease in node impurity averaged over all trees of the ensemble.

2. The "***sum of squared coefficients***", where each coefficient is the output of a binary **linear-kernel support vector machine (SVM)** classifier (i.e., one-vs-one classifier or one-vs-rest classifier) for a given feature. Usually, the squared coefficient of a feature learnt by a binary SVM is intuitively regarded as this feature's importance and has been widely adopted for ranking and selecting features [5,6]. In the multi-class problem which is often solved by constructing a series of binary SVM classifiers (i.e., one-vs-one classifier or one-vs-rest classifier) to build a multi-class SVM classifier, we can easily sum up the squared coefficients of a given feature learnt from this series of binary SVM classifers for quantifying the importance of this feature in the multi-class problem.

Both feature importance measures have already been implemented in "scikit-learn" package. For the simplicity of notation, we term them as **gini-importance feature selection method** and **SVM-coefficient feature selection method**. Detailed parameters of these two feature selection methods are listed below.

- **Parameters of gini-importance feature selection method:** The only parameter is the number of trees, which we chose from the set of 8 integers $\{2^3, 2^4, 2^5, 2^6, 2^7, 2^8, 2^9, 2^{10}\}$. Therefore, we have 8 different parameter combinations for this method.

- **Parameters of SVM-coefficient feature selection method:** Binary SVM classifiers (that are used for building the multiclass classifier) are built using two schemes, i.e., one-vs-one and one-vs-rest. The penalty parameter of the binary SVM classifier is chosen from a set of 11 values $\{2^{-5}, 2^{-3}, 2^{-1}, 2^1, 2^3, 2^5, 2^7, 2^9, 2^{11}, 2^{13}, 2^{15}\}$. Taken together, we have 22= 2×11 different parameter combinations for this method.

In addition, in both feature selection methods, we select the top- k features (ranking by feature importance scores) from the full feature set (i.e., 42,374 features), where $k \in \{500, 1000, 2000, 4000, 8000, 16000, 32000\}$. These result in 210= $(8 + 22)\times 7$ sets of selected features, where each feature set is selected according to a method, a parameter combination, and a k value.

After obtaining these 210 feature sets, we then use them to compare the prediction performance of RF and SVM multi-class prediction methods on each of these 210 feature sets. The parameters of the RF and SVM multi-class prediction methods use the same parameter combination sets as aforementioned in feature selection step. Particularly for the SVM multi-class prediction method, we use not only linear kernel, but also radial-basis-function (RBF) kernel with its gamma parameter chosen from the set of 10 values $\{2^{-15}, 2^{-13}, 2^{-11}, 2^{-9}, 2^{-7}, 2^{-5}, 2^{-3}, 2^{-1}, 2^1, 2^3\}$ .Therefore, the RF multi-class prediction method has 8 different parameter combinations and the SVM multi-class prediction method has 242=$2\times 11\times(1 + 10)$ different parameter combinations. Taken together with the 210 sets of features selected, we can evaluate and compare the performance of each multi-class prediction method with a feature set and a parameter combination. In total, we have 1680=210×8 prediction results for the RF multi-class method and 50820=210×242 prediction results for the SVM multi-class method.

C. Prediction results on simulation data with low (10%) or high (50%) level of copy number aberration (CNA) events

We repeat the analysis on simulation data with different frequencies of CNA events. Let p_c be the probability that the copy number of a CpG cluster is c. The probabilities of different copy numbers are $p_0 = 0.002, p_1 = 0.053, p_2 = 0.9, p_3 = 0.035, p_4 = 0.008, p_5 =$

0.002, when the overall frequency of CNA events (i.e., $p_0 + p_1 + p_3 + p_4 + p_5$) is set to 10%. These probabilities of are set as $p_0 = 0.008, p_1 = 0.266, p_2 = 0.5, p_3 = 0.178, p_4 = 0.04, p_5 = 0.008$, for the simulation with a high level (50%) of CNA events.

Using the simulation data with 10% of CNA events, the Pearson's correlation coefficient (PCC) and root mean squared error (RMSE) between true and predicted ctDNA burden (θ) are 0.973 and 0.076, respectively. When more (50%) CNA events are added to the simulation data, PCC and RMSE remain the same (0.973 and 0.076, respectively). Therefore, the level of CNA events does not show a strong effect on the performance of tumor burned prediction. Figure S1 A and B show the estimated ctDNA burdens for the simulated normal samples and cancer-patient plasma samples with 10% CNA events, respectively. The corresponding results on dataset with 50% CNA events are given in Figure S3 C and D. Please note that, although no CNA event is simulated for normal plasma samples, the results illustrated in Figure S1 A and C are slightly different because the optimized likelihood cutoffs are different, which is used to determine whether a sample is from a cancer patient or not.

As shown in Figure S2, CancerLocator achieves almost identical performance on datasets with fewer (10%) or more (50%) CNA events. In addition, CancerLocator outperforms both RF and SVM methods on plasma samples with small ctDNA burdens. This is consistent with the results reported in the main text, where the frequency of CNA events in the simulation is 30%. The detailed prediction performance of the three methods on the two additional simulation datasets are listed in Table S1.

Taken together, CancerLocator demonstrates a robust performance against copy number aberration.

A

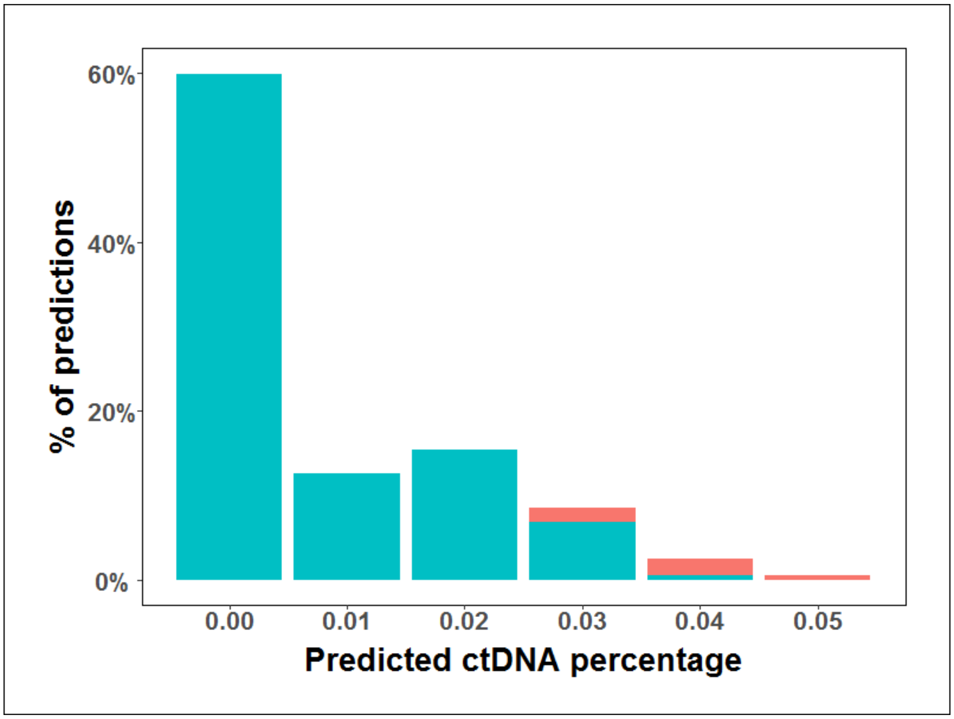
60%
40%
20%
0%
% of predictions
0.00
0.01
0.02
0.03
0.04
0.05
Predicted ctDNA percentage

B

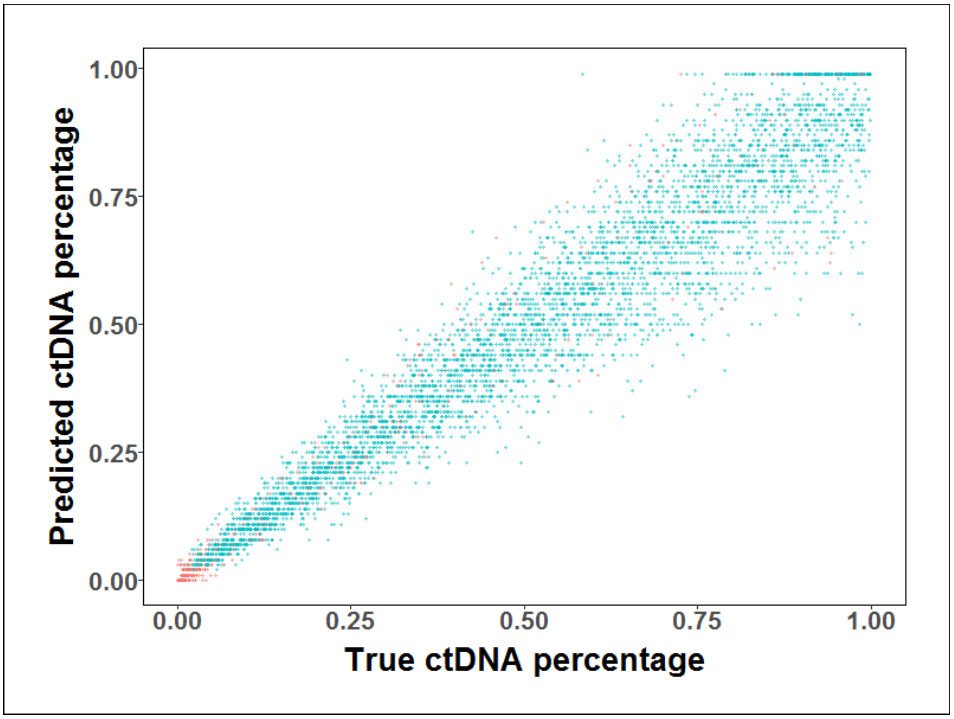
1.00
0.75
0.50
0.25
0.00
Predicted ctDNA percentage
0.00
0.25
0.50
0.75
1.00
True ctDNA percentage

C

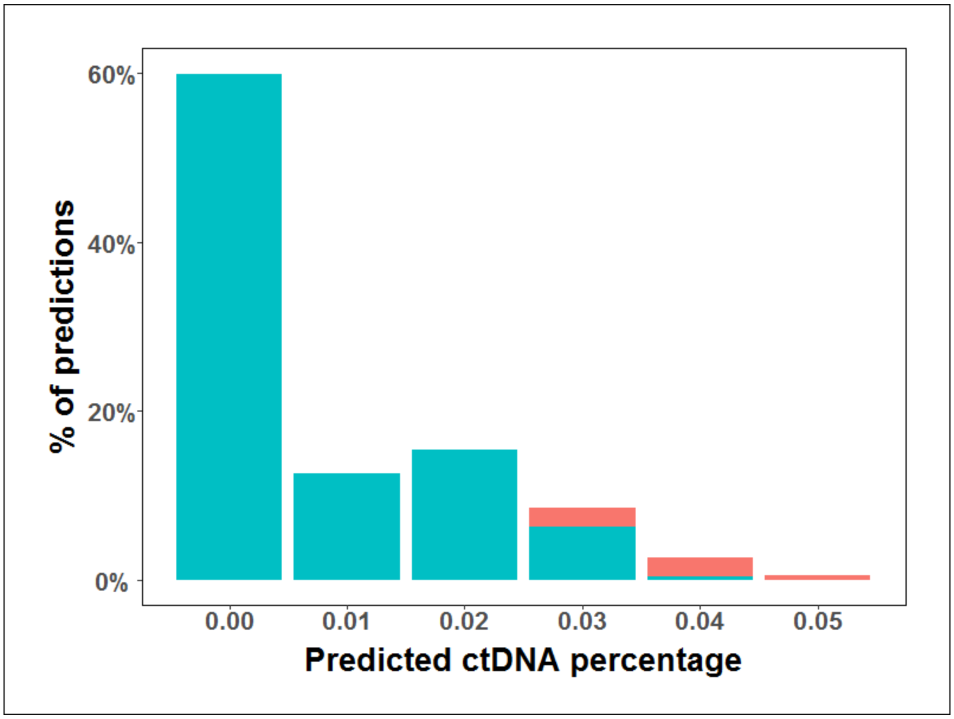

D

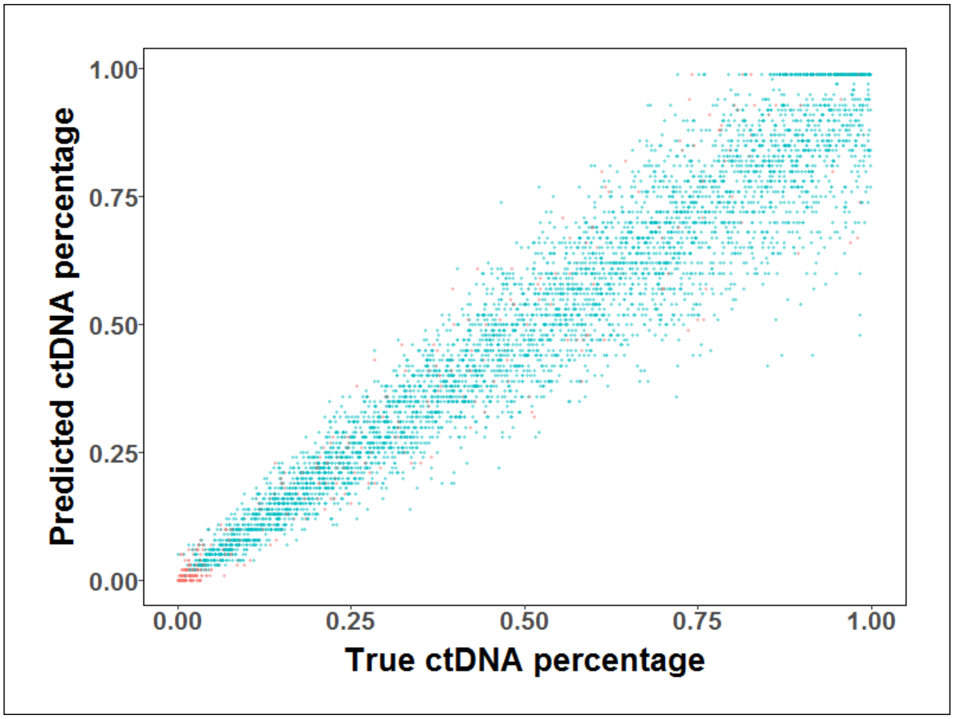

Figure S1. The predicted ctDNA burdens for simulated normal samples and cancer samples with 10% (A&B) or 50% (C&D) CNA events. (A&C) The histogram of predicted ctDNA burdens for normal samples. **(B&D)** The predicted and true ctDNA burdens for cancer samples. Each dot represents a prediction with the true (x-axis) and predicted (y-axis) ctDNA burdens. The correct and incorrect predictions are represented by cyan and red, respectively.

A

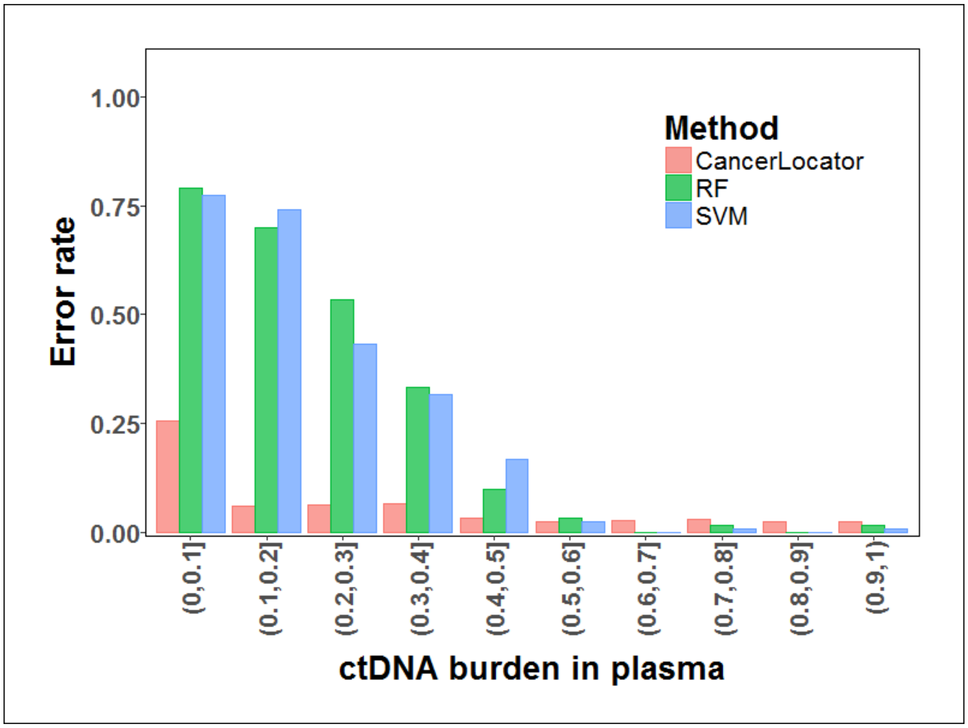

B

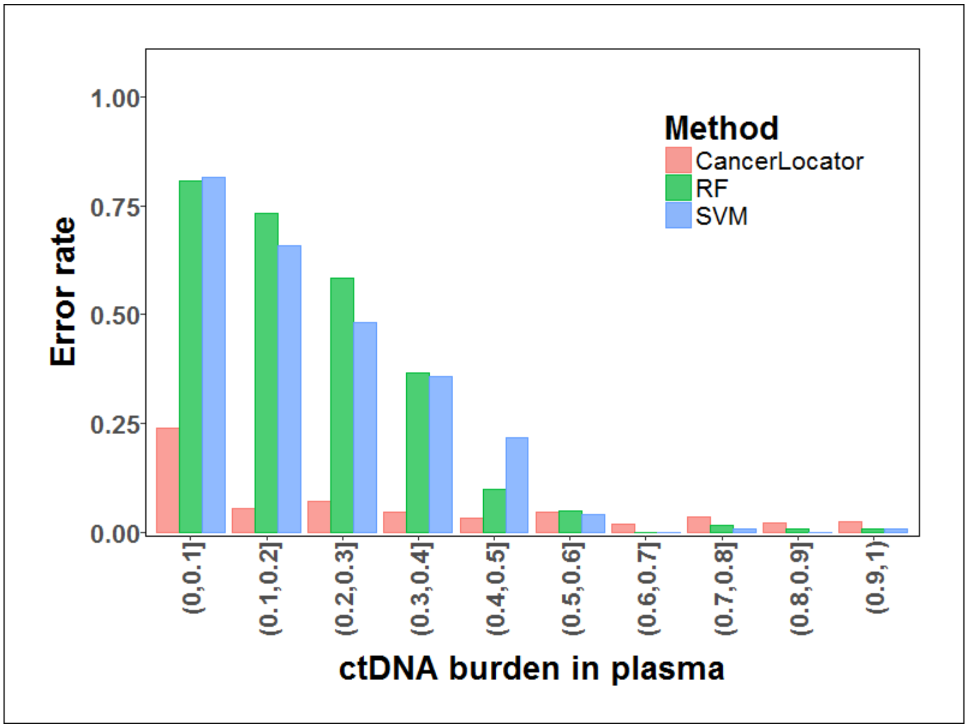

Figure S2. Classification performances of three methods (CancerLocator, RF and SVM) on the ten subsets of simulation data. Each subset includes cancer plasma cfDNA samples at certain cancer stage (represented as a ctDNA burden range). The frequency of CNA events is set to 10% **(A)** or 50% **(B)** in the two simulations.

Table S1. The error rate of predictions on the two additional simulation datasets of different frequencies of CNV events.

		The frequency of CNA events in simulation	
		Low level of CNA (10%)	High level of CNA (50%)
Method	CancerLocator	0.074	0.074
	RF	0.289	0.295
	SVM	0.291	0.328

Table S2. Demographic and clinical features of the patients enrolled in this study.

**Lab ID**	**Final Diagnosis**	**Clinical Stage (Lung Cancer) or Benign**
948	Adenocarcinoma	Stage 1A; T1aN0
954	Adenocarcinoma	
955	Adenocarcinoma	Stage 1B; T2aN0
962	Adenocarcinoma	Stage 1B; T2aN0
964	Non-small cell carcinoma	Stage IIIA/IV
978	Fibroelastotic scar - negative for malignancy	Benign
982	Right lower lobe squamous cell carcinoma	
987	Adenocarcinoma	Stage 1B; T2aN0
989	Adenocarcinoma	Stage 1A; T1bN0

References

1. Chan KCA, Jiang P, Chan CWM, Sun K, Wong J, Hui EP, et al. Noninvasive detection of cancer-associated genome-wide hypomethylation and copy number aberrations by plasma DNA bisulfite sequencing. Proc. Natl. Acad. Sci. U. S. A. 2013;110:18761–8.

2. Pedregosa F, Varoquaux G, Gramfort A, Michel V, Thirion B, Grisel O, et al. Scikit-learn: Machine Learning in Python. J. Mach. Learn. Res. 2011;12:2825–30.

3. Breiman L. Random Forests. Mach. Learn. Kluwer Academic Publishers; 2001;45:5–32.

4. Menze BH, Kelm BM, Masuch R, Himmelreich U, Bachert P, Petrich W, et al. A comparison of random forest and its Gini importance with standard chemometric methods for the feature selection and classification of spectral data. BMC Bioinformatics. 2009;10:213.

5. Guyon I, Weston J, Barnhill S, Vapnik V. Gene Selection for Cancer Classification using Support Vector Machines. Mach. Learn. Kluwer Academic Publishers; 2002;46:389–422.

6. Zhou X, Tuck DP. MSVM-RFE: extensions of SVM-RFE for multiclass gene selection on DNA microarray data. Bioinformatics. 2007;23:1106–14.
